# Mitochondrial pathophysiology beyond the retinal ganglion cell: occipital GABA is decreased in autosomal dominant optic neuropathy

**DOI:** 10.1007/s00417-018-4153-z

**Published:** 2018-10-15

**Authors:** Otília C. d’Almeida, Inês R. Violante, Bruno Quendera, Miguel Castelo-Branco

**Affiliations:** 1 Visual Neuroscience Laboratory, Institute for Biomedical Imaging and Life Sciences (CNC.IBILI), Faculty of Medicine, University of Coimbra, Azinhaga de Santa Comba, 3000-548 Coimbra, Portugal; 2 Coimbra Institute for Biomedical Imaging and Translational Research (CiBIT), Institute for Nuclear Sciences Applied to Health (ICNAS), University of Coimbra, Coimbra, Portugal; 3 School of Psychology, Faculty of Health and Medical Sciences, University of Surrey, Guildford, UK

**Keywords:** Autosomal dominant optic neuropathy, GABA, Glutamate, Neurotransmission, Retinal ganglion cell

## Abstract

**Purpose:**

It has remained a mystery why some genetic mitochondrial disorders affect predominantly specific cell types such as the retinal ganglion cell. This is particularly intriguing concerning retinal and cortical function since they are tightly linked in health and disease. Autosomal dominant optic neuropathy (ADOA) is a mitochondrial disease that affects the ganglion cell. However, it is unknown whether alterations are also present in the visual cortex, namely in excitation/inhibition balance.

**Methods:**

In this study, we performed in vivo structural and biochemical proton magnetic resonance imaging in 14 ADOA and 11 age-matched control participants focusing on the visual cortex, with the aim of establishing whether in this genetically determined disease an independent cortical neurochemical phenotype could be established irrespective of a putative structural phenotype. Cortical thickness of anatomically defined visual areas was estimated, and a voxel-based morphometry approach was used to assess occipital volumetric changes in ADOA. Neurochemical measurements were focused on γ-aminobutyric acid (GABA) and glutamate, as indicators of the local excitatory/inhibitory balance.

**Results:**

We found evidence for reduced visual cortical GABA and preserved glutamate concentrations in the absence of cortical or subcortical atrophy. These changes in GABA levels were explained by neither structural nor functional measures of visual loss, suggesting a developmental origin.

**Conclusions:**

These results suggest that mitochondrial disorders that were previously believed to only affect retinal function may also affect cortical physiology, especially the GABAergic system, suggesting reduced brain inhibition vs. excitation. This GABA phenotype, independent of sensory loss or cortical atrophy and in the presence of preserved glutamate levels, suggests a neurochemical developmental change at the cortical level, leading to a pathophysiological excitation/inhibition imbalance.

**Electronic supplementary material:**

The online version of this article (10.1007/s00417-018-4153-z) contains supplementary material, which is available to authorized users.

## Introduction

Several neurodegenerative, neuromuscular, and inherited diseases are strongly related to both mitochondrial dysfunction and optic nerve atrophy [1]. Concerning the latter, optic neuropathies are frequently associated with chronic visual impairment that may lead to blindness in both pediatric and adult populations [2]. In fact, the high-energy requirements of the neural visual pathway increase the likelihood of excessive oxidative stress and neural damage when mitochondrial dysfunction occurs.

Autosomic dominant optic neuropathy (ADOA, Kjer’s disease, MIM no. 165500) is one of the most common hereditary mitochondrial diseases with an estimated prevalence set between 1:12,000 (Denmark, founder effect) and 1:50,000 [3]. ADOA has a progressive degeneration profile that “commences as a degeneration of retinal ganglion cells, with secondary ascending optic atrophy and changes of the corresponding tract and areas in the lateral geniculate body” [4].

Phenotypically, this disorder is characterized by moderate to severe central vision deterioration and/or blindness due to optic nerve atrophy and retinal nerve fiber layer (RNFL) degeneration [5]. Despite the well-documented expression of ADOA as an isolated optic nerve pathology [6], less is known regarding the impact on the brain. In fact, several patients develop extraocular complications such as neurosensory hearing loss, myopathy, peripheral neuropathy, chronic progressive external ophtalmoplegia being commonly named “plus” phenotypes [7]. These syndromic forms highlight the high vulnerability of the peripheral and possibly also the central nervous system (CNS) to degeneration and dysfunction in ADOA.

Genetic studies have associated this disorder to pathogenic mutations mainly within the nuclear gene OPA1 in chromosome 3q28-q29 that encodes for inner mitochondrial membrane proteins [8]. These mutations have an impact on the subunit I of the mitochondrial transport chain leading to the disruption of mitochondrial dynamics and influencing the normal mitochondrial fusion and fission balance [6, 9].

A previous study showed that the OPA1 gene is ubiquitously expressed in both retina, optic tract, and brain [10] rendering quite intriguing the assumption of the predominant selectivity of impairment of retinal ganglion cells (RGCs) in genetic mitochondrial disorders. Interestingly, the smaller-caliber RGCs within the inner retina constituting the papillomacular bundle are particularly vulnerable while melanopsin-containing RGCs are relatively spared [2].

The retina and the brain are closely related and share embryological, anatomical, and physiological features [11]. It is therefore important to understand whether changes in the retina are correlated with alterations in cortical and subcortical structures and whether they are independent. In our previous study of preclinical Leber Hereditary Optic Neuropathy (LHON) carriers, we found higher cortical thickness values in the peripheral visual extrastriate representations and these changes were predicted by the swelling of macular RGC axons [12, 13]. Other authors have also established retinocortical-associated changes in healthy aging, cognitive impairment and Alzheimer’s disease [14], and in other pathologies such as multiple sclerosis [15], glaucoma [16], and in patients with long-lasting retinal visual defects [17] in both anatomical and functional domains [18].

Also, disturbances in the excitation/inhibition balance in cortical neural networks have been implied not only in several disorders such as autism spectrum disorders and schizophrenia [19] but also during cortical development [20] and plasticity processes [21]. The maintenance of this balance, mainly through the regulation of glutamate (excitatory) and GABA (inhibitory) levels, is pivotal for normal brain physiology.

This is the first study investigating both structural and excitation/inhibition cortical phenotypes of ADOA, a condition that leads to the loss of central vision with optic nerve atrophy and retinal ganglion cell degeneration. More specifically, motivated by the hypothesis that visual cortex neurochemistry and structure might be altered due to the fact that the OPA1 gene is expressed in the brain, we performed cortical thickness and volumetric analysis, and also in vivo neurochemical analysis using proton MR spectroscopy (^1^H-MRS) in the occipital cortex of a group of OPA1-ADOA patients from a previously studied cohort in a vision research study [22] to (indirectly) assess neurotransmission.

## Materials and methods

### Participants

We tested 14 ADOA patients (5 males, mean age ± SD = 35.8 ± 17.50 years) belonging to different pedigrees (Supplementary material, Table S1). These participants are a subgroup from a cohort described elsewhere [22]. The diagnosis was clinically consistent with ADOA and confirmed by genetic testing whenever a mutation could be found (see Supplementary material, Table S1, for details). Participants from the ADOA group were submitted to MRI acquisition, and data were compared to an age-matched [*t*(23) = 0.417, *p* = 0.681] control group (11 participants; 5 males, mean age ± SD = 33.3 ± 10.84 years). All participants were checked for the presence of other neuro-ophthalmologic pathology, besides ADOA. Control participants had normal visual acuities (VA, equal or superior to 1.0) while ADOA patients had low VA (mean ± SD = 0.3 ± 0.16). ADOA patients were also submitted to functional and structural assessment using the Color Cambridge Test (CCT), the pattern Electroretinogram (pattern ERG), and Spectralis Optic Coherence Tomography (OCT) testing as described in [13]. For each test, the ADOA group was compared to an independent age- and gender-matched control group (Supplementary material, Fig. S1).

Exclusion criteria for controls included retinal, neurologic, or psychiatric disease under medication, diabetes even in the absence of retinopathy, retinal significant media opacities, pseudophakic and aphakic eyes, and high ammetropies (sphere > ± 4D; cylinder > ± 2D). The study followed the tenets of the Declaration of Helsinki, and was approved by the local Institutional Review Board. Informed consent was obtained from each patient after procedures of the research had been fully explained.

### MRI data acquisition

**Fig. 1:**
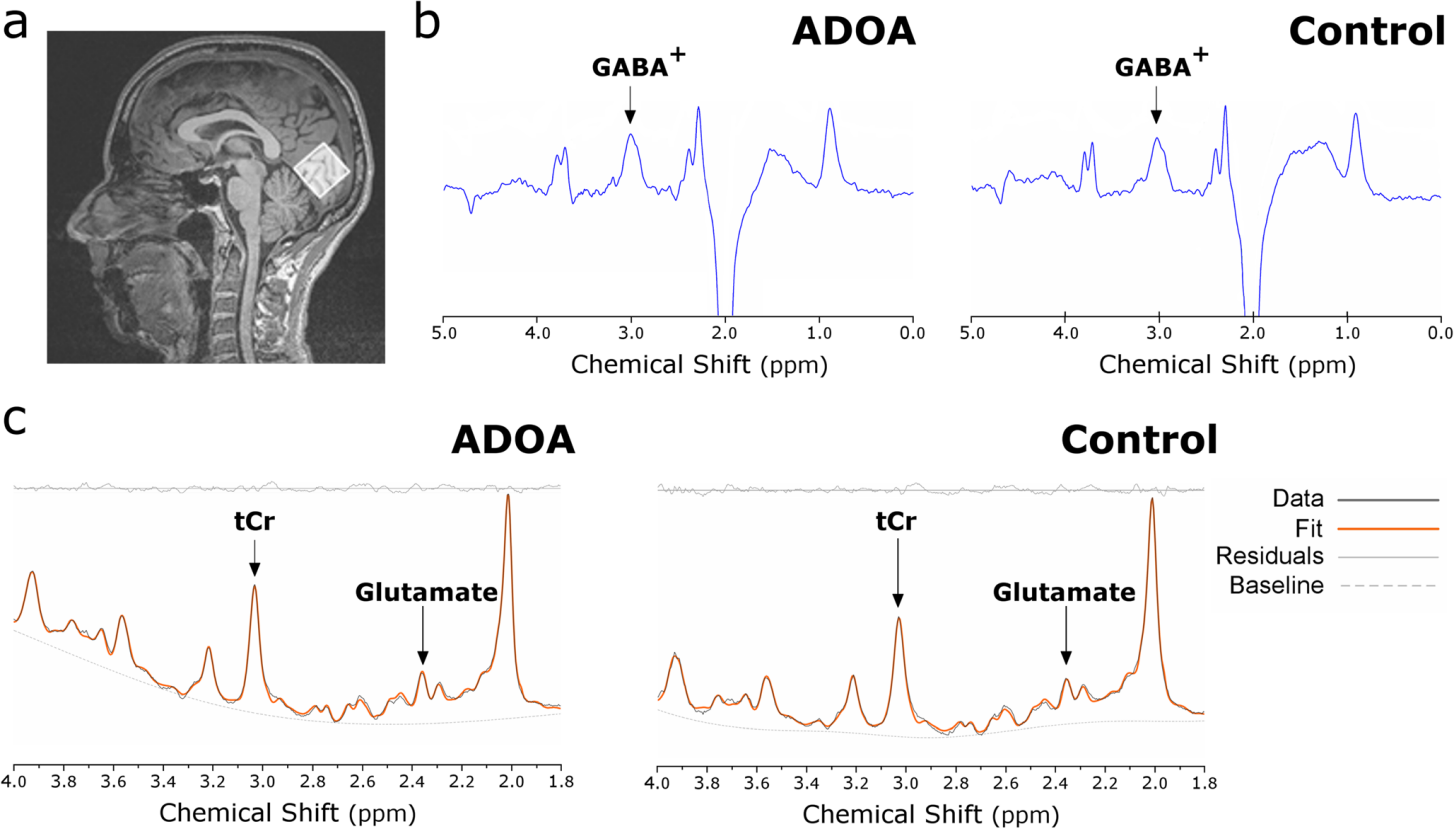
Spectroscopic acquisition and data processing. Sagittal view of a **a** representative magnetic resonance spectroscopy voxel acquired in the gray matter-rich occipital lobe. **b** GABA^+^ levels were estimated through ^1^H-MRS MEGA-PRESS sequence and analyzed through Gannet. PRESS spectroscopy data **c** was analyzed through LCModel to estimate glutamate levels. The representative spectroscopy data are from the same ADOA patient (Supplementary table, patient no. 3, male, 19 years) and control (male, 20 years). In **c**, it is shown the processed data (black solid line), the LCModel fitted spectrum (orange solid line), the residuals (gray solid line, on top), and the baseline (dashed gray line). *tCr*, total creatine

In this study, we acquired MRI data in a 3T scanner (Siemens Magnetom TrioTim 3 T Erlangen, Germany), with a 12-channel head coil. For each participant, two three-dimensional Magnetization Prepared Rapid Acquisition Gradient Echo (MPRAGE) sequences were acquired (repetition time (TR) 2.3 s, echo time (TE) 2.98 ms, flip angle (FA) 9°, field of view (FoV) 256 × 256 mm^2^, yielding 160 slices with 1 × 1 × 1 mm^3^ voxel size). ^1^H-MRS was performed in a 3 × 3 × 3 cm^3^ voxel positioned medially in the occipital cortex (Fig. 1a). To avoid lipid contamination from the outer edges of the cerebrum, the voxel inevitably may have encompassed to a limited extent some cortical matter extraneous to the striate cortex. A Point RESolved Spectroscopy (PRESS, TE = 30 ms, TR = 2 s, 160 averages, 1024 data points) sequence was used to estimate glutamate levels. The MEshcher-GArwood Point RESolved Spectroscopy (MEGA-PRESS, TE = 68 ms, TR = 1.5 s, 1024 data points) sequence was used to specifically estimate the levels of GABA. During odd number acquisitions (196 averages), a frequency-selective inversion pulse was applied to the GABA-C3 resonance at 1.9 ppm (ON) and during even number acquisitions (196 averages), the pulse was applied at 7.5 ppm (OFF).

### MRI data analysis

All image processing and morphometric procedures to estimate cortical thickness were performed with BrainVoyager QX 2.8 (Brain Innovation, Maastricht, The Netherlands). Structural data pre-processing was similar to the described elsewhere [12, 23]. To improve statistical group-level analysis and to use anatomically defined regions of interest (ROI) from an atlas, a cortical-based alignment procedure was performed. This procedure enhances the quality of the alignment between brains, using the curvature information of the cortex. Cortical thickness was estimated using an automatic algorithm that creates intermediate equipotential smoothed surfaces between two different intensity values, i.e., between the inner, white matter–gray matter (WM–GM), and outer, GM-cerebrospinal fluid (CSF) boundaries, by applying the second-order partial differential Laplace’s equation [24]. The sum of all small steps executed along the field lines between the two surfaces gives a thickness value for each point. After the computation, a cortical thickness map was superimposed in the volumetric data file, and then interpolated into 3D spherical cortical representations. Using BVQX tools, ROIs were overlaid on the cortical thickness maps and the mean values of thickness for each area were calculated. To prevent outlier biases, an outlier removal criterion was considered. This approach consisted in the recalculation of the mean thickness excluding the values deviating more than 3 standard deviations (SD) from the mean.

On the other hand, volumetric analyses were conducted using SPM8 (Wellcome Trust Centre for Neuroimaging, Institute of Neurology, UCL, London, UK, http://www.fil.ion.ucl.ac.uk/spm/) and VBM8 toolboxes (http://dbm.neuro.uni-jena.de/vbm8/) running in Matlab (MATLAB R2013a, TheMathworks, USA) environment. Briefly, the following steps were followed: (1) two MPRAGE images were aligned and averaged to increase the signal-to-noise ratio (SNR); (2) the image was manually centered to the anterior commissure (AC) and re-aligned onto the AC-posterior commissure (PC) axis; (3) through VBM8, the image was automatically corrected for field inhomogeneities, normalized to MNI reference space, and segmented into three main tissues based on probabilistic maps, GM, WM, and CSF through the Diffeomorphic Anatomical Registration using the Exponentiated Lie algebra (DARTEL) algorithm [25]; (4) since we were examining volume changes, GM and WM images were modulated to scale for the expansions and contractions performed during spatial normalization; (5) modulated images were smoothed with a Gaussian kernel of 8 mm (FWHM).

We used a standard, independent *t* test with an absolute threshold masking of 0.1 to investigate structural changes (including both GM and WM volumes) between ADOA and control groups in SPM8 software. Statistical inferences were made at 0.05 significance level (corrected for multiple comparisons using Family Wise Error (FWE)).

### ^1^H-MRS data analysis

To measure GABA levels more accurately, we used a J-difference editing technique (MEGA-PRESS) [26]. MEGA-PRESS data were analyzed using Gannet GABA-MRS Analysis Tool [27] version 2.0 for MATLAB (R2013a, v.8.1.0, TheMathWorks, USA). Three hertz exponential line broadening was applied to all spectra prior to the Fast Fourier Transform of the time resolved data. After the frequency and phase correction and the pairwise outlier rejection of data for which frequency correction fitting parameters were greater than three standard deviations from the mean, the edited difference spectrum was generated for each dataset (Fig. 1b). Gannet uses nonlinear least-squares fitting to integrate the ~ 3.00 ppm of both GABA (Gaussian model applied in the difference spectrum) and creatine (Lorentzian model applied in the OFF spectrum). MEGA-PRESS has been the standard technique to measure in vivo GABA signal. By removing overlapping contributions of other metabolites, this difference-editing approach returns a GABA signal, more robust than PRESS GABA signal, but with contributions of macromolecules signals. Therefore, GABA signal will be referred herein as GABA^+^.

PRESS spectra were analyzed using the LCModel version 6.3 [28] using a linear combination of prior knowledge in vitro standard basis set (Fig. 1c). All spectra were visually inspected. Crámer-Rao Lower Bounds (CRLB) for glutamate were less than 6%. Spectra were fitted between 4 and 1.8 ppm to avoid contamination from lipids at lower frequencies. GABA^+^ Glutamate levels were normalized to the total creatine + phosphocreatine (tCr) signal to reduce inter-subject variability (GABA^+^/tCr and Glu/tCr, respectively).

### Statistical analysis

All statistical analyses were performed with IBM SPSS Statistics 22 for Windows (version 22, IBM Corp., Armonk, NY, USA). Parametric independent *t* tests were performed to compare ROI thicknesses and metabolite ratios between groups. Whenever the normality assumption was not met (Shapiro-Wilk test, *p* ≤ 0.05), Mann-Whitney tests were used instead. Regarding spectroscopy data analysis, the values of Glu/tCr and GABA^+^/tCr that fell outside the interquartile range (IQR) multiplied by a factor of 2.2 were considered as outliers and removed from analysis [29]. Two-tailed hypothesis tests were performed at a 0.05 significance level.

## Results

### ^1^H-magnetic resonance spectroscopy analysis

The fractions of gray matter (GM), white matter (WM), and cerebrospinal fluid (CSF) enclosed in the acquired voxel were estimated using an in-house MATLAB script relying on the SPM8-VBM8 toolboxes. No differences in tissue content were found between ADOA and control groups (Table 1).

**Table 1:** Mean tissue fraction for ADOA and control groups

	Tissue fraction (mean ± SD)		*p* value
	ADOA	Control	
*N*	14	11	–
GM (%)	67.2 ± 5.59	70.3 ± 6.89	0.238
WM (%)	16.7 ± 4.91	17.0 ± 4.13	0.873
CSF (%)	13.9 ± 3.95	12.1 ± 5.06	0.329

**Fig. 2:**
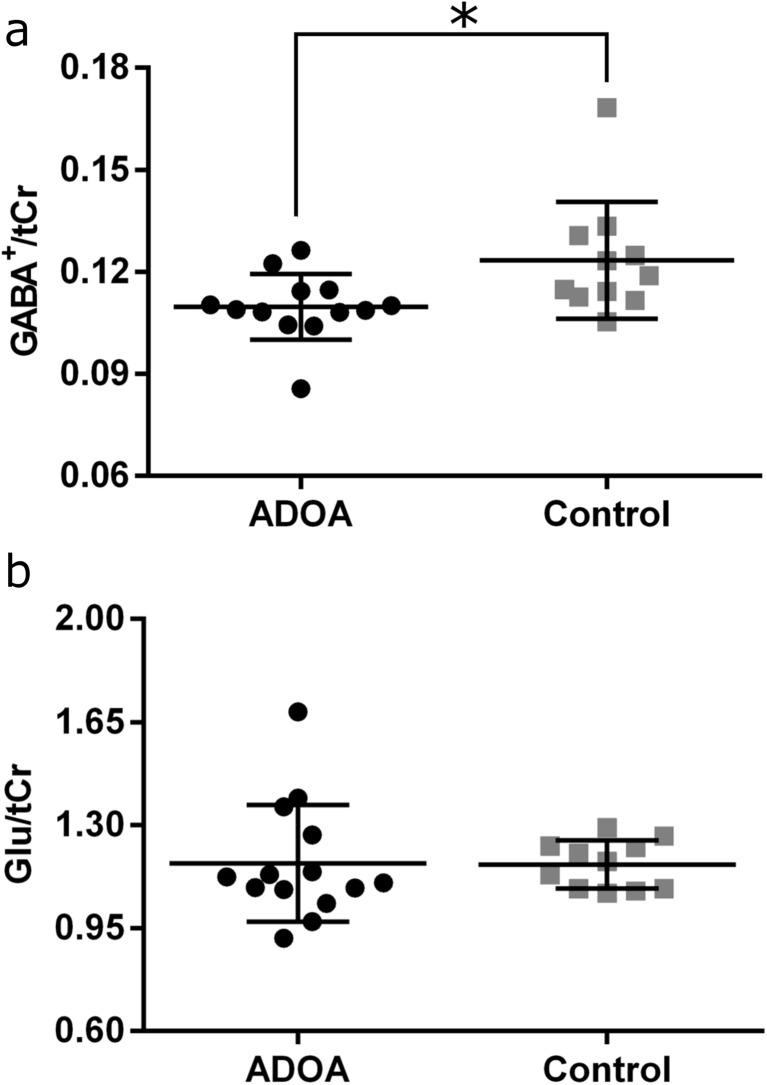
Proton magnetic resonance spectroscopy analysis. **a** GABA^+^/total creatine (tCr) and **b** Glu/tCr levels for both groups (ADOA, black circles; Controls, gray squares). *p = 0.011. Graphs depict individual values, mean and standard deviation

We acquired MEGA-PRESS and PRESS data to measure respectively free GABA and glutamate levels in the occipital cortex of both ADOA patients and healthy controls (Figs. 1 and 2). We found a marked decrease for GABA^+^/tCr ratio [*N* = 24, Mann-Whitney *Z* = −2.525, *p* = 0.011] in ADOA patients, compared to healthy controls while no differences were found in Glu/tCr ratio [*t*(23) = − 0.285, *p* = 0.778]. Extreme value inspection retrieved an outlier for GABA^+^/tCr in an ADOA participant. Still, after removing this value, the group analysis results were similar [GABA^+^/tCr ratio: *N* = 23, Mann-Whitney *Z* = − 2.339, *p* = 0.019].

As a secondary analysis, we performed bivariate Pearson (and Spearman, as appropriate) correlations between GABA^+^/tCr and all visual function and structural assessment variables depicted in Supplementary material, for ADOA cohort. There were no significant correlations between GABA^+^/tCr levels and any of the ophtalmologic variables documenting sensory loss collected in the ADOA group.

### MRI structural analysis

#### Cortical thickness analysis

We estimated mean cortical thickness in anatomically defined occipital areas and both pre- and post-central gyri of both hemispheres. Occipital areas comprised Brodmann areas 17, 18, and 19, as a proxy for primary visual area V1, extrastriate areas V2/V3, and higher-level areas, respectively, and also several relevant gyri (cuneus, precuneus and inferior, middle and superior occipital gyri). No significant group differences were found for any of the analyzed regions.

#### VBM analysis

**Fig. 3:**
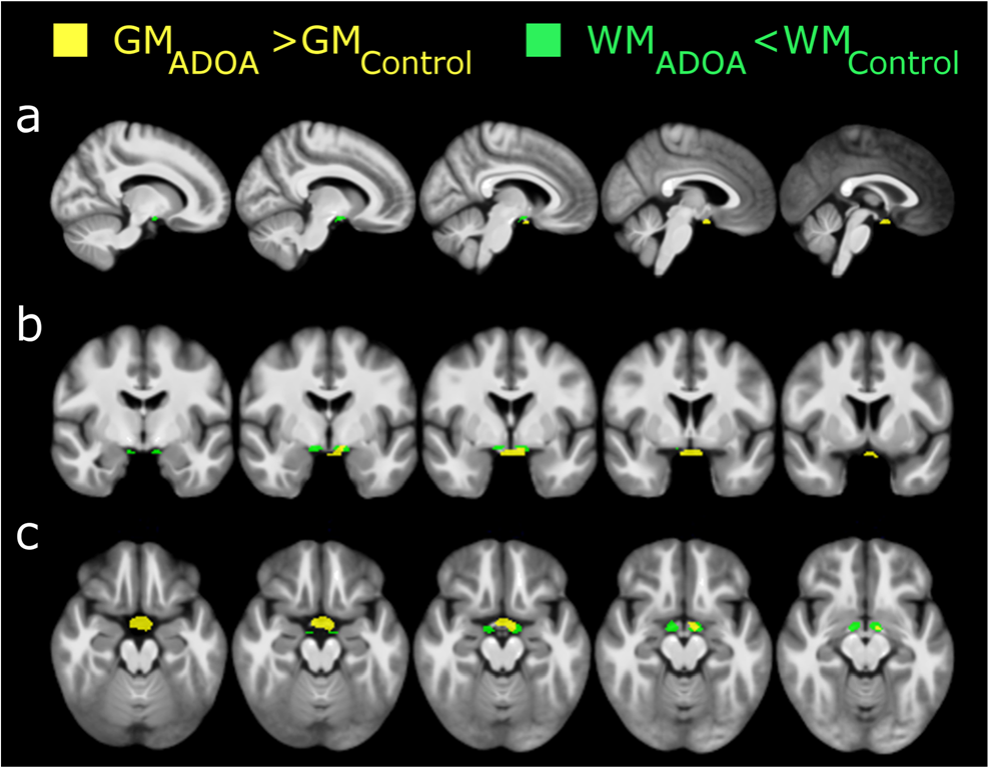
Whole-brain voxel-based morphometry analysis between ADOA patients and healthy controls. Results of VBM analysis presented at a voxel-level *p* value < 0.001, uncorrected (only for visualization purposes), with smoothing FWHM = 8 cm^3^ on **a** sagittal, **b** coronal, and **c** axial MRI slices. Binarized masks showing significant GM (yellow) and WM (green) relative volume differences in the visual pathway are overlaid on an average image of all participants. Note that neither cortical nor subcortical regions seem to be affected, and alterations are present only in chiasmatic regions (after correction for multiple comparisons)

We performed whole-brain voxel-based morphometry to examine white matter volumetric differences between the ADOA patients and controls in the visual tract. Two clusters showed reduced volume in WM images of ADOA patients compared to controls in post-chiasma structures [cluster 1: peak *T* = 6.60; *Z* = 4.89; x,y,z = 11, − 3, − 17; *p*_FWEcorr_ = 0.014; cluster 2: peak *T* = 7.11; *Z* = 5.12; x, y, z = − 9, − 3, − 17; *p*_FWEcorr_ = 0.005] (Fig. 3).

## Discussion

In this work, we investigated the hypothesis that cortical neurochemistry and in particular inhibitory balance is impaired in patients with genetically determined mitochondrial dysfunction. We report the first cortical morphometry analysis and in vivo measurements of glutamate and GABA in patients with OPA1-ADOA. We found a significant decrease of GABA^+^/tCr levels in the occipital lobe of ADOA patients, in contrast with preserved glutamate levels, suggesting a functional decrease in cortical inhibition. Structural measures of volumetric VBM and cortical thickness did not reveal changes in the occipital cortex of these patients.

OPA1-ADOA is a rare disease associated with point mutations in the OPA1 gene ubiquitously expressed that is crucial for mitochondrial function regulation. In this disease, the central papillomacular bundle is especially affected due to the retinal ganglion cell (RGC) degeneration leading to optic nerve atrophy and ensuing central visual field loss related to mitochondrial dysfunction [30].

In our recent work with asymptomatic Leber Hereditary Optic Neuropathy (LHON) individuals, also an inherited mitochondrial optic neuropathy, we found evidence for enhanced developmental mechanisms of cortical plasticity in visual extrastriate cortex [12]. We also found that this regionally specific cortical reorganization is triggered by changes in macular retinal ganglion cell axonal layer thickness [13], suggesting a retinal cause. We did not find structural cortical changes in the occipital lobe of ADOA patients, evaluated with both cortical thickness and volumetric measures, as based on the anatomical definition of our ROIs. Our results are consistent with the findings of Rocca and colleagues [31] who used VBM and Tract-Based Spatial Statistics (TBSS) to assess regional GM and WM changes in ADOA patients. They found significant WM atrophy of the chiasm and optic tract but no atrophy of GM regions.

In spite of the absence of volumetric changes, we found independent changes in cortical biochemistry. A previous study with OPA1 patients revealed several brain imaging abnormalities but normal creatine, *N*-acetylaspartate, and choline levels [32], suggesting preserved metabolism. However, neurotransmitter levels were not accessed. Indeed, our hypothesis was based on the tenet that impaired mitochondrial functioning can cause alterations in neurotransmission [33] in this disorder. Also, it is possible that intracortical reorganization mechanisms may be counteracting in the visual cortex against retinal degeneration processes. A shift in excitatory/inhibitory balance is consistent with this idea. A developmental origin for our findings is corroborated by the absence of a relation with structural (OCT) and functional markers of visual loss (psychophysical, as indexed by visual acuity and color testing along cone contrast axes; and electrophysiological, as indexed by pattern ERG).

In our study, we focused the changes in excitatory/inhibitory balance in OPA1 patients, as assessed by the quantification of glutamate and GABA. First and foremost, glutamate is the main excitatory neurotransmitter of the CNS. Glutamate effects are short-lived and its concentration needs to be strictly controlled due to excitotoxic effects. Excessive excitatory neurotransmission can be triggered by mitochondrial dysfunction, oxidative stress, calcium overload, and energy deficiency, ultimately leading to neurodegeneration [34, 35]. In our study, glutamate levels were not different between patients and controls.

On the other hand, GABA is a surrogate marker of inhibitory neurotransmission, and it is commonly associated with brain function as assessed from neurophysiology and the behavioral points of view [36–38]. GABA level changes have been implicated in the pathophysiology of several neurologic disorders [39], brain maturation [40], neuroplasticity events [41, 42], and recovery post-stroke [43].

As a cautionary note, in vivo spectroscopy does not allow the discrimination between intracellular or extracellular GABA, i.e., the metabolic (cytosolic) GABA or the neurotransmitter (vesicular) (GABA). In any case, the decrease of GABA in this mitochondrial disorder may be linked with disrupted Ca^2+^ homeostasis that occurs in ADOA due to OPA1 loss [44, 45] leading to changes in neurotransmitter release [46].

Also, the physiological impact of this specific change in the excitatory/inhibitory balance may possibly be related to plasticity-related developmental phenomena [47]. As stated above, the fact that there is no correlation of GABA/tCr with visual dysfunction (VA, OCT, CCT, and pattern ERG) renders less likely a mechanism based on lack of sensory input.

The pathophysiological implications of the observed lower levels of GABA in the visual cortex, in the absence of volumetric changes, should be further explored from the mechanistic point of view. In this line, lowering of GABA levels is possibly linked with modulation by factors such as insulin-like growth factor 1 (IGF1) [48], which has been suggested to be a potentially effective therapy for mitochondrial protection and recovery [49].

Future studies, with larger cohorts and complementary neuroimaging techniques might be helpful in elucidating the nature of putative homeostatic mechanisms, for example by measuring GABA-A receptor binding potential which could be addressed by positron-emission tomography (PET) imaging.

We provide evidence for impaired cortical physiology (reduced inhibition vs. excitation as assessed by reduced GABA levels with preserved glutamate levels) with structural sparing in ADOA, given the identified GABAergic changes in the visual cortex. These results suggest a novel cortical physiological alteration that may be relevant for the exploration of hitherto unexpected brain dysfunction of this retinal ganglion cell disorder. Future studies should address the impact of this novel phenotype on visual and cognitive function, as identified in other neurodegenerative disorders.

## Electronic supplementary material

ESM 1 (DOCX 209 kb)
