# Supplementary material for: Mitochondrial pathophysiology beyond the retinal ganglion cell: occipital GABA is decreased in autosomal dominant optic neuropathy
Source: Graefes Arch Clin Exp Ophthalmol. 2018 Oct 15;256(12):2341–8. doi: 10.1007/s00417-018-4153-z (PMC6224020; doi:10.1007/s00417-018-4153-z)
Supplement: Supplementary file 1 — (DOCX 209 kb) [file 417_2018_4153_MOESM1_ESM.docx]

**Mitochondrial pathophysiology beyond the retinal ganglion cell: occipital GABA is decreased in autosomal dominant optic neuropathy**

**Supplementary Data**

**Table S1** Demographic and genetic characteristics of the ADOA participants. *LE*, left eye; *RE*, right eye.

| **ID** | **Gender** | **Age (y)** | **Family** | **Mutation** | |
| --- | --- | --- | --- | --- | --- |
|  |  |  |  | **at coding DNA level** | **at protein level** |
| 1 | F | 56 | D | c.2708_2711del | p.Val903Glyfs*3 |
| 2 | F | 16 | A | c.869 G>A | p.Arg290Gln het |
| 3 | M | 19 | A | c.869 G>A | p.Arg290Gln het |
| 4 | F | 40 | A | c.869 G>A | p.Arg290Gln het |
| 5 | F | 33 | I | No mutation identified |  |
| 6 | F | 48 | B | c.2708_2711del | p.Val903Glyfs*3 |
| 7 | F | 76 | B | c.2708_2711del | p.Val903Glyfs*3 |
| 8 | M | 19 | C | c.2131 C>T | p.Arg711*het |
| 9 | M | 39 | F | No mutation identified |  |
| 10 | F | 41 | F | No mutation identified |  |
| 11 | M | 31 | L | No mutation identified |  |
| 12 | M | 47 | D | c.2708_2711del | p.Val903Glyfs*3 |
| 13 | F | 16 | H | No mutation identified |  |
| 14 | F | 20 | K | No mutation identified |  |

**Ophtalmologic Evaluation**

To briefly evaluate the ophthalmologic status of the ADOA cohort, visual acuity (Table S1) was measured and Color Cambridge Test (CCT), pattern Electroretinogram (pattern ERG) and Spectralis Optic Coherence Tomography (OCT) (Figure S1) were performed as described in (Mateus *et al.*, 2016). Statistical analyses were performed with IBM SPSS Statistics 22 for Windows (version 22, IBM Corp., Armonk, NY, USA). For each test, ADOA group was compared to an independent age- and gender-matched control group. Group comparisons were made using independent t-tests whenever normality assumptions (Shapiro-Wilk test). Otherwise, Mann-Whitney tests were used instead. Each variable represent the mean value of both eyes for each participant. Two-tailed hypothesis tests were performed at a 0.05 significance level.


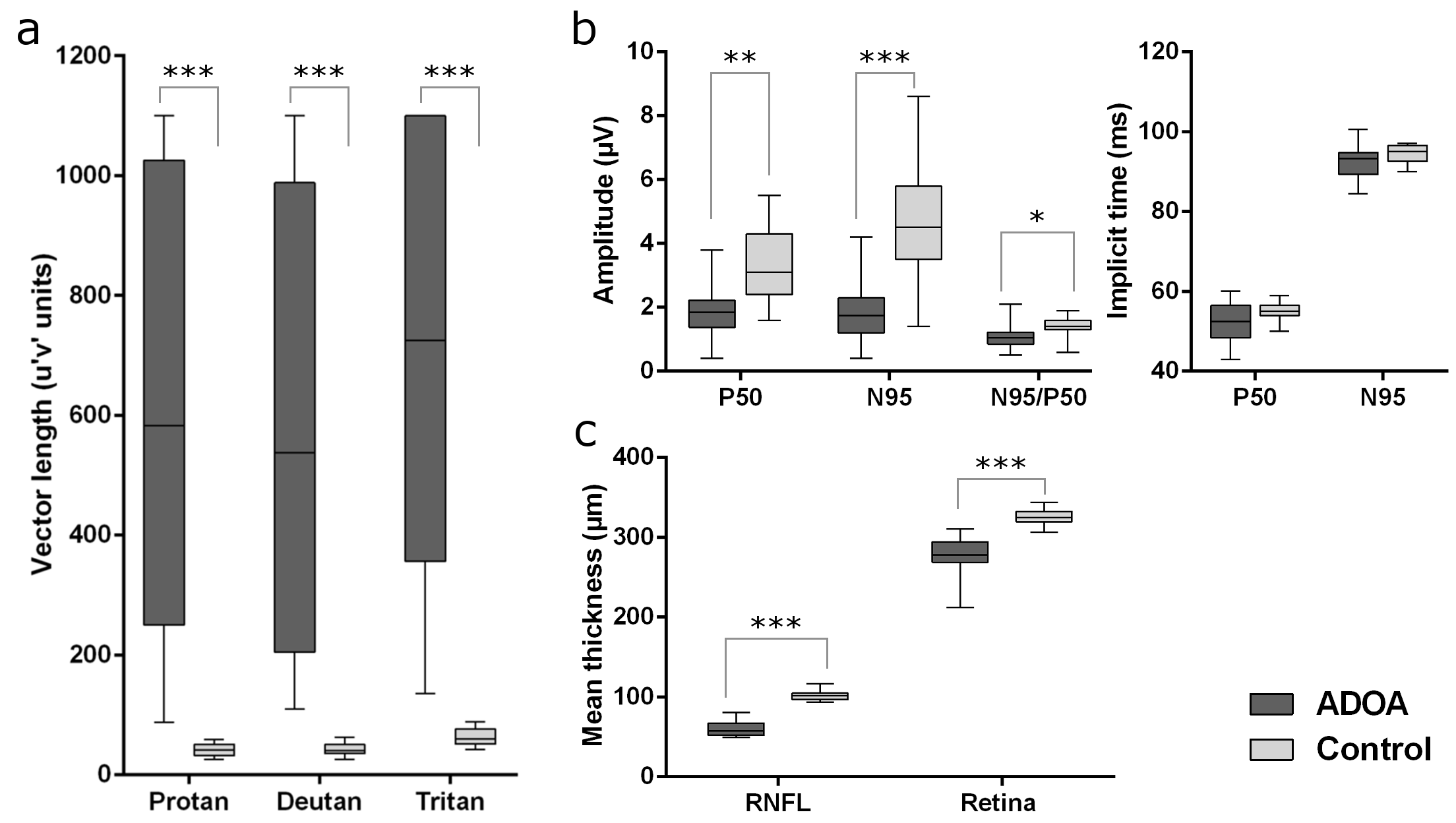


**Figure S1.** To briefly evaluate the ophthalmologic status of the ADOA cohort (N=14, male: 35.7%, mean age±SD=35.8±17.5), **(a)** Color Cambridge Test (CCT), **(b)** pattern Electroretinogram (PERG) and **(c)** Spectralis Optic Coherence Tomography (OCT) were performed. For each test, ADOA group was compared to an independent age- and gender-matched control group. **(a) *Cambridge Color Test*** (Control group: N=19, male: 21.1%, mean age±SD=34.1±13.2): There was a severe impairment for all three chromatic axes as the participants with ADOA had significantly higher thresholds when compared with controls (*Protan*: t(13.014)=5.553, p<0.0001; *Deutan*: Z=-4.845, p<0.0001; *Tritan*: t(13.030)=6.238, p<0.0001); **(b) *pattern ERG*** (Control group: N=19, male: 31.6%, mean age±SD=36.5±17.1): ADOA group had significant lower P50 and N95 waves amplitudes (*P50 amplitude*: t(31)=-3.911, p<0.001; *N95 amplitude*: t(31)=-5.316, p<0.0001). We also found a significant decrease of the N95/P50 ratio (Z=-4.845, p=0.001). However no differences were found for the P50 and N95 implicit times; **(c) *Optic Coherence Tomography*** (Control group: N=12, male: 41.7%, mean age±SD=39.3±15.9): Mean thickness was defined as the average thickness from all 9 regions of Early Treatment Diabetic Retinopathy Study (ETDRS) map. Retinal Nerve Fiber Layer (RNFL) and retina thickness were, as expected, significantly inferior in ADOA group compared to controls (RNFL: t(24)=-11.689, p<0.001; retina: t(24)=-6.345, p<0.001). ***p<0.0001; **p<0.001; *p=0.001
